# Association Between White Matter Tract Microstructure and Cognitive, Affective, and Somatic Empathy in Children: An Exploratory TRACULA‐Based Diffusion MRI Study

**DOI:** 10.1002/brb3.71751

**Published:** 2026-09-20

**Authors:** June Christoph Kang

**Affiliations:** ^1^ School of Psychology and Clinical Language Sciences University of Reading Reading UK; ^2^ Department of Brain and Cognitive Engineering Korea University Seoul South Korea; ^3^ Empathy Research Institute Goyang South Korea

**Keywords:** CASES, children, diffusion MRI, empathy, fractional anisotropy, TRACULA, white matter

## Abstract

**Purpose:**

Empathy is a multidimensional construct encompassing cognitive, affective, and somatic components, yet its white matter correlates have been examined primarily in adults and typically using measures limited to cognitive and affective dimensions. This study explored associations between white matter tract microstructure and multidimensional trait empathy in typically developing children.

**Methods:**

Twenty‐six typically developing children (14 boys, 12 girls; mean age 10.1±2.2 years, observed range 7–15) underwent diffusion MRI. White matter pathways were reconstructed with TRACULA‐based probabilistic tractography, and trait empathy was assessed with the Korean version of the Cognitive, Affective, and Somatic Empathy Scales (CASES‐K). Along‐tract general linear models with within‐tract permutation‐based cluster correction (5000 sign‐flip permutations) tested associations between fractional anisotropy (FA) and empathy scores, controlling for age and sex.

**Findings:**

At within‐tract correction, three clusters showed negative FA–empathy associations: lower FA in the left superior longitudinal fasciculus I (SLF1; *p* = 0.032) and the genu of the corpus callosum (*p* = 0.048) with higher total empathy, and lower FA in the left SLF3 with higher affective empathy (*p* = 0.020). However, no clusters survived correction across the full set of 168 tract–subscale analyses (all false‐discovery‐rate *q* ≈ 1.0), so the findings are best interpreted as exploratory and hypothesis‐generating. Within these clusters, the negative FA associations coincided with higher radial diffusivity and essentially unchanged axial diffusivity, and remained after controlling for in‐scanner motion.

**Conclusion:**

These preliminary results are consistent with the possibility that different empathy dimensions relate to partially distinct white matter pathways during childhood. They require replication in larger samples, however, before any mechanistic, maturational, or causal interpretation is warranted.

## Graphical abstract

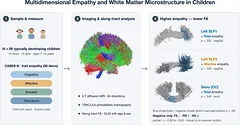

## 1 Introduction

Empathy—the capacity to understand and share the emotional states of others—is a fundamental aspect of human social cognition that enables adaptive interpersonal functioning (Decety and Jackson 2004; Singer and Lamm 2009). Historically, empathy was conceptualized as a unitary construct. However, accumulating theoretical and empirical evidence has established that empathy is a multidimensional phenomenon comprising dissociable components (Davis 1983; Blair 2005; Batson 2009). Blair (2005) proposed three distinct forms of empathy: cognitive empathy, which involves understanding another person's mental state or perspective; affective empathy, which entails sharing another person's emotional experience; and somatic (motor) empathy, which refers to the automatic tendency to mimic others’ bodily expressions and postures. This tripartite model has been supported by evidence from both typical and clinical populations, with different psychiatric conditions exhibiting selective impairments in specific empathy components (Blair 2005).

Despite the theoretical recognition of empathy as a multifaceted construct, its measurement long remained limited to instruments assessing only cognitive and affective dimensions, such as the widely used Interpersonal Reactivity Index (Davis 1983). To address this gap, Raine and Chen (2018) developed the Cognitive, Affective, and Somatic Empathy Scales (CASES), a 30‐item self‐report instrument for children and adolescents that measures all three empathy components, with additional differentiation between positive and negative affective valence within each dimension. Confirmatory factor analysis supported a three‐factor structure, and the scale demonstrated good internal consistency (Cronbach's α=0.91), criterion validity, and predictive validity (Raine and Chen 2018). The CASES has been cross‐culturally validated, including the Korean version (CASES‐K); that validation confirmed the three‐factor structure and demonstrated satisfactory psychometric properties in Korean elementary school children (Kim and Choi 2020).

The neural underpinnings of empathy have been investigated extensively in social and affective neuroscience. Functional neuroimaging studies have identified a distributed network of regions supporting empathic processes, including the anterior insula, anterior cingulate cortex, inferior frontal gyrus, temporoparietal junction, and medial prefrontal cortex (Decety and Jackson 2004; Singer and Lamm 2009). However, the contribution of structural brain connectivity—particularly the white matter pathways that provide the anatomical infrastructure for communication between these regions—has received comparatively less attention.

White matter microstructure has increasingly been linked to empathic functioning. Diffusion tensor imaging (DTI), which measures the anisotropic diffusion of water along axonal fibers, provides indices of white matter microstructure such as fractional anisotropy (FA), mean diffusivity (MD), axial diffusivity (AD), and radial diffusivity (RD) (Basser et al. 1994; Le Bihan et al. 2001). These metrics are sensitive but biologically nonspecific: They are jointly influenced by axon density, myelination, fiber diameter, fiber geometry, and partial‐volume effects, and should not be equated with white matter “integrity” (Beaulieu 2002; Jones et al. 2013). Several key pathways have nonetheless been implicated in empathy. The uncinate fasciculus (UF), connecting orbitofrontal cortex with the anterior temporal lobe and amygdala, has been linked to emotional empathy; lesion work indicates that acute damage to the right UF impairs emotional empathy performance (Oishi et al. 2015). The superior longitudinal fasciculus (SLF), linking prefrontal regions with the temporoparietal junction, has been associated with cognitive empathy and perspective‐taking (Wang et al. 2018; Veerareddy et al. 2025). The cingulum bundle, connecting cingulate cortex with medial temporal structures, has been related to mentalizing and affective processing (Wang and Olson 2018). The inferior longitudinal fasciculus (ILF) and corpus callosum have additionally been linked to emotion recognition and interhemispheric integration of affective information (Comes‐Fayos et al. 2018).

Tract‐specific investigations suggest that white matter microstructure tracks individual differences in empathic capacity, although the direction of these associations is not uniform. Parkinson and Wheatley (2014) reported that emotional empathy (Interpersonal Reactivity Index empathic concern) was *positively* associated with FA in limbic and perception–affect pathways. Steinberg et al. (2022), by contrast, found that FA in the corpus callosum, corticospinal tract, SLF, and inferior fronto‐occipital fasciculus was negatively associated with empathy scores in healthy young females. Veerareddy et al. (2025) demonstrated that FA in the left SLF was positively correlated with social network size, with cognitive empathy mediating the relationship between left cingulum microstructure and social connectedness. A review by Comes‐Fayos et al. (2018) concluded that the tracts most consistently associated with empathy are the UF, SLF, ILF, and inferior fronto‐occipital fasciculus. The heterogeneity of reported directions indicates that FA–empathy associations do not have a simple, fixed biological interpretation.

Several gaps remain. First, most studies have measured empathy with instruments capturing only cognitive and affective dimensions, omitting somatic empathy. Second, whole‐tract tractography approaches—rather than region‐of‐interest analyses—remain underutilized in empathy research. Third, studies in children are scarce, despite childhood being a critical period for both empathy development and white matter maturation (Lebel and Beaulieu 2012). TRACULA (TRActs Constrained by UnderLying Anatomy) is an automated global probabilistic tractography tool that reconstructs major pathways using anatomical priors from training data within a Bayesian framework (Yendiki et al. 2011), yielding reproducible volumetric pathway distributions without manual intervention. The method has been updated with ultra‐high‐gradient Human Connectome Project training data, enabling automated reconstruction of 42 pathways from routine‐quality diffusion data (Maffei et al. 2021).

Given the small, cross‐sectional sample available, the present study was explicitly designed as an exploratory, hypothesis‐generating investigation of associations between TRACULA‐reconstructed white matter microstructure and multidimensional trait empathy (CASES‐K) in children. By combining a measure that captures cognitive, affective, and somatic components with automated reconstruction of major pathways, we sought to identify candidate structure–behavior relationships to be tested in future, adequately powered studies.

## 2 Methods

### 2.1 Participants

Twenty‐eight typically developing children were enrolled from the community in the Seoul metropolitan area, South Korea. Two participants were excluded because usable diffusion MRI data were not available (incomplete or unprocessable diffusion acquisition), yielding a final analytic sample of 26 children (14 boys, 12 girls). Eligibility criteria were: (1) age between 6 and 16 years, (2) no history of neurological or psychiatric disorders, (3) no contraindications for MRI, and (4) right‐handedness. The observed age range of the analytic sample was 7–15 years (mean =10.08±2.15 years; median =10). Written informed consent was obtained from all parents or legal guardians, and verbal assent was obtained from each child prior to participation. The study was approved by the Institutional Review Board of Korea University Guro Hospital (approval number: 2021GR0275).

### 2.2 Measures

#### 2.2.1 Korean Cognitive, Affective, and Somatic Empathy Scales

Trait empathy was assessed using the Korean version of the CASES‐K, validated by Kim and Choi (2020) based on the original CASES (Raine and Chen 2018). The CASES‐K is a 30‐item self‐report questionnaire measuring three dimensions of empathy: cognitive empathy (10 items), affective empathy (10 items), and somatic empathy (10 items), each further divided into positive and negative affective valence subscales (5 items each). Items are rated on a three‐point Likert scale (0 = *rarely*, 1 = *sometimes*, 2 = *often*), with higher total scores indicating greater empathic capacity. The CASES‐K has demonstrated good internal consistency (Cronbach's α=0.92 for the total scale; 0.80–0.87 for subscales) and a confirmed three‐factor structure in Korean children (Kim and Choi 2020).

### 2.3 MRI Data Acquisition

MRI data were acquired at Korea University Guro Hospital using a Siemens 3 T Magnetom Prisma scanner (Siemens Healthineers, Erlangen, Germany) with a 32‐channel head coil. T1‐weighted anatomical images were obtained using a magnetization‐prepared rapid gradient‐echo (MPRAGE) sequence: repetition time (TR) = 2300 ms; echo time (TE) = 2.32 ms; inversion time = 900 ms; flip angle = 8°; field of view (FOV) = 230 mm; voxel size = 0.9 mm isotropic; 208 sagittal slices; GRAPPA acceleration factor = 2; receiver bandwidth = 200 Hz/pixel; echo spacing = 7.1 ms.

Whole‐brain diffusion‐weighted images (DWI) were acquired using a single‐shot spin‐echo echo‐planar imaging sequence with 64 non‐collinear gradient directions (b=1000  s/mm 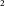) and one non‐diffusion‐weighted volume (b=0 s/mm 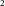), with two signal averages (90 volumes total). Additional parameters were: TR = 3100 ms; TE = 78 ms; flip angle = 90°; acquisition matrix = 112 × 112; FOV = 224 mm; voxel size = 1.0 × 1.0 × 2.0 mm 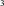.

### 2.4 Structural MRI Processing

Cortical reconstruction and volumetric segmentation were performed using FreeSurfer (version 7.4.1; http://surfer.nmr.mgh.harvard.edu/) (Fischl 2012). The pipeline included motion correction, non‐brain tissue removal, automated Talairach transformation, subcortical and white matter segmentation, intensity normalization, gray–white boundary tessellation, topology correction, and surface deformation. Cortical parcellations followed the Desikan–Killiany atlas (Desikan et al. 2006). All segmentations were visually inspected in Freeview by a trained rater who was blind to participants’ empathy scores, and manual corrections were applied when necessary.

### 2.5 White Matter Tractography

Automated probabilistic reconstruction of white matter pathways was performed using TRACULA, as implemented in FreeSurfer 7.4.1 (Yendiki et al. 2011; Maffei et al. 2021). TRACULA uses a Bayesian framework for global tractography that incorporates anatomical priors from a training atlas (Jbabdi et al. 2007) and employs the ball‐and‐stick diffusion model, which allows multiple fiber orientations per voxel (Behrens et al. 2003, 2007).

#### 2.5.1 Diffusion Preprocessing and Motion Correction

TRACULA preprocessing (*trac‐all ‐prep*) corrected the diffusion data for eddy‐current–induced distortions and head motion using FSL *eddy* (Jenkinson et al. 2012; Andersson and Sotiropoulos 2016), which simultaneously models off‐resonance and subject movement and performs slice‐wise outlier detection and replacement. The DWI were then registered to the T1‐weighted anatomy, and within‐voxel orientation distributions were estimated with *bedpostx*. Pathway reconstruction (*trac‐all ‐path*) performed Bayesian global tractography for each of the 42 major pathways, and tract‐specific diffusion measures were extracted with *trac‐all ‐stat*.

#### 2.5.2 Quality Control and Motion Assessment

For each participant, we summarized in‐scanner motion from the *eddy*/TRACULA motion outputs: average volume‐to‐volume translation, average rotation, the percentage of slices flagged as signal dropout (“bad”), and the mean total root‐mean‐square (RMS) movement. Motion was uniformly low across the sample (mean average translation =0.11±0.04 mm, range 0.06–0.21; mean average rotation =0.0017±0.0013°; mean bad‐slice fraction =0.02%, maximum 0.25%; mean RMS movement =0.52±0.34 mm). No participant exceeded conventional motion thresholds, and no additional volumes were removed beyond the slice‐wise outlier replacement performed internally by *eddy*. In addition, all 42 reconstructed tracts were visually inspected following an established quality‐control protocol (He et al. 2021) by a rater blind to empathy scores; no reconstruction failures or partially reconstructed tracts were identified. As a sensitivity analysis, we confirmed that the reported associations were unchanged when average translation was added as a covariate (see Section 3).

For each of the 42 pathways, along‐tract profiles and tract‐averaged values of FA, MD, AD, and RD were computed as weighted averages over the pathway's posterior probability distribution. FA indexes the directional coherence of diffusion, AD the diffusivity along the principal direction, RD the diffusivity perpendicular to it, and MD the overall diffusivity magnitude.

### 2.6 Statistical Analysis

#### 2.6.1 Behavioral Analyses

Descriptive statistics (mean, SD, range) were computed for the CASES‐K total and for the cognitive, affective, and somatic subscales. Pearson correlations quantified relationships among subscales and between each empathy score and age. Sex differences were tested with Welch's *t*‐tests (effect sizes as Cohen's *d*). These analyses characterized the sample and assessed whether age or sex structured the empathy measures.

#### 2.6.2 Along‐Tract Imaging Model

Associations between white matter microstructure and empathy were examined with along‐tract general linear models (GLMs) using FreeSurfer's *mri_glmfit*. Each tract was represented by its standard TRACULA along‐tract profile (equidistant cross‐sectional sampling points spanning the pathway; e.g., 70 points for the SLF3 pathway), and the per‐point diffusion value was the cross‐sectionally weighted average over the pathway's posterior. For each pathway, FA at each along‐tract point was modeled as a function of the empathy score using a different‐offset–different‐slope (DODS) design, with separate intercepts and slopes for boys and girls and with age included as a continuous nuisance covariate. The contrast of interest tested the average (sex‐pooled) empathy slope, that is, [00000.50.5] over the design columns (female intercept, male intercept, female age, male age, female empathy, male empathy). FA was the primary metric because it is the most widely reported tract‐microstructure index, maximizing comparability with prior empathy DTI studies (Parkinson and Wheatley 2014; Steinberg et al. 2022; Veerareddy et al. 2025); MD, AD, and RD were retained for the supportive analyses described below. Separate models were fitted for the CASES‐K total and for each of the three subscales, yielding 42×4=168 tract–subscale analyses.

#### 2.6.3 Multiple‐Comparison Correction

Within each tract, family‐wise error across along‐tract points was controlled non‐parametrically using cluster‐wise inference (FreeSurfer *mri_glmfit‐sim*/*mri_volcluster*). A cluster‐forming threshold of p<0.05 ($|-\mathrm{lo}g_{10}p|>1.3$) was applied to the signed significance map, and cluster‐wise p‐values (CWP) were derived from the null distribution of maximum cluster size over 5000 sign‐flip permutations of the contrast. Because the analysis comprised 168 such tract–subscale GLMs, we additionally controlled error across the *entire* analytic search space: The most significant CWP from each analysis was carried forward and corrected across all 168 analyses using both the Benjamini–Hochberg false discovery rate (Benjamini and Hochberg 1995) and Bonferroni procedures. Clusters significant only at the within‐tract level are reported as exploratory.

#### 2.6.4 Supportive, Effect Size, and Concordance Analyses

To characterize effect magnitudes and to examine whether the FA findings were corroborated by the other diffusion metrics, we extracted, for each significant cluster, the participant‐wise mean FA, MD, AD, and RD averaged across the cluster's along‐tract points (using the cluster membership map produced by *mri_volcluster*). Partial correlations between each cluster‐mean metric and the relevant empathy score were computed controlling for age and sex (and, in a sensitivity analysis, additionally for average translation), with 95% confidence intervals via the Fisher z transform. These cluster‐mean analyses, and the scatterplots in Figure 2, are presented for visualization and effect‐size description only; because the clusters were selected for their FA association, the cluster‐mean FA correlations are statistically non‐independent of that selection and are reported descriptively rather than as independent confirmatory tests.

## 3 Results

### 3.1 Sample Characteristics and Empathy Scores

The analytic sample comprised 26 children (14 boys, 12 girls; mean age 10.08±2.15 years, range 7–15). Age was distributed across the range (7 y: n=3; 8 y: n=5; 9 y: n=3; 10 y: n=3; 11 y: n=5; 12 y: n=5; 14 y: n=1; 15 y: n=1). Descriptive statistics for the CASES‐K total and subscales and the inter‐subscale correlations are reported in Table 1. Total empathy ranged from 20 to 56 (mean 40.85±9.06). The subscales were moderately to strongly intercorrelated, except for the cognitive–affective correlation, which was weak and non‐significant (r=0.32, p=0.12), consistent with partial separability of these dimensions.

**TABLE 1:** Descriptive statistics for CASES‐K empathy scores and inter‐subscale Pearson correlations (N=26).

			Correlations (r)			
Scale	Mean (SD)	Range	Cog.	Aff.	Som.	Total
Cognitive	14.35 (3.87)	5–20	—	0.32	0.67 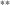	0.82 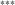
Affective	14.54 (3.58)	6–19	0.32	—	0.55 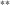	0.75 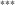
Somatic	11.96 (3.59)	5–19	0.67 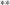	0.55 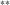	—	0.90 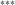
Total	40.85 (9.06)	20–56	0.82 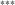	0.75 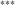	0.90 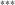	—

*Note*: The cognitive–affective correlation was not significant (p=0.12).

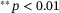
.

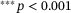
.

Empathy scores were not significantly associated with age (total r=0.25, p=0.21; cognitive r=0.30, p=0.14; affective r=0.19, p=0.36; somatic r=0.13, p=0.53), indicating that the empathy measures were not strongly structured by the developmental age span. For sex, affective empathy was higher in girls than in boys (16.17±3.10 vs. 13.14±3.46; t=−2.35, p=0.027, d=0.92); cognitive (p=0.78), somatic (p=0.50), and total (p=0.24) empathy did not differ significantly by sex, and age did not differ by sex (p=0.85). All imaging models controlled for both age and sex.

### 3.2 Along‐Tract Associations Between FA and Empathy

**FIGURE 1:**
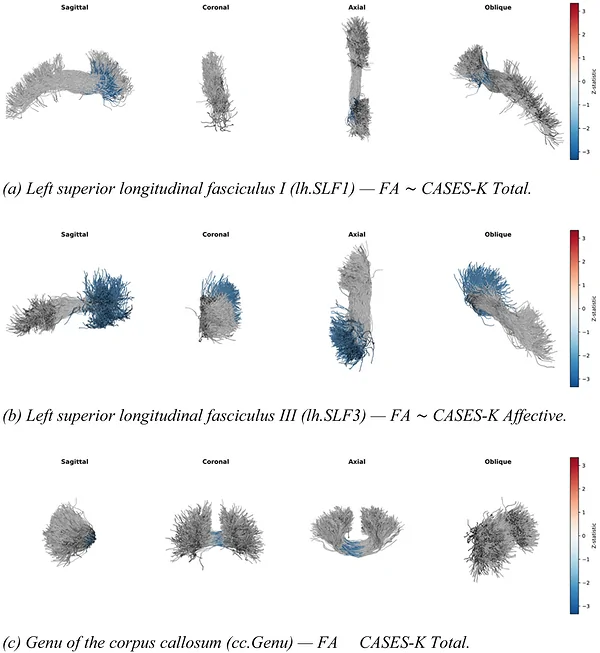
Along‐tract visualization of the three exploratory clusters showing negative associations between fractional anisotropy (FA) and CASES‐K empathy scores, from four viewing angles (sagittal, coronal, axial, oblique). Tracts are colored by the along‐tract Z‐statistic (blue: negative; red: positive). Non‐significant segments are gray; significant clusters (within‐tract permutation p<0.05) are saturated. (a) lh.SLF1 × Total (p=0.032); (b) lh.SLF3 × Affective (p=0.020); (c) cc.Genu × Total (p=0.048). None survived correction across all 168 analyses.

**FIGURE 2:**
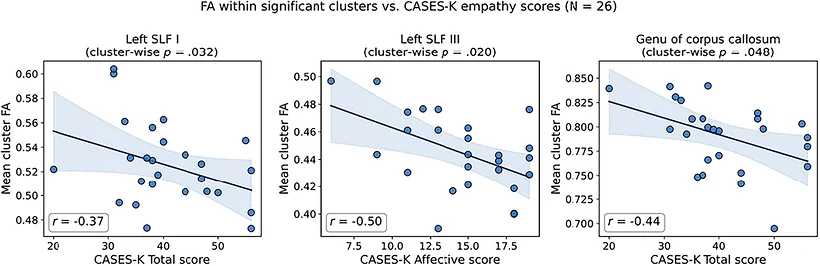
Cluster‐mean FA versus CASES‐K empathy scores for the three exploratory clusters (N=26). Panels, left to right: left SLF1 versus total empathy; left SLF3 versus affective empathy; genu of the corpus callosum versus total empathy. Lines show the linear fit; shaded bands are 95% confidence intervals; r values are zero‐order Pearson correlations of the cluster‐mean FA. These plots are provided for visualization and effect‐size description only. Because each cluster was selected for its FA association, the displayed FA correlations are not independent of that selection and should not be read as confirmatory effect estimates.

Across the 168 tract–subscale analyses, exactly three analyses yielded an along‐tract cluster significant at the within‐tract permutation‐corrected level; all three were negative associations (higher empathy associated with lower FA), and no positive clusters were found in any analysis (Table 2; Figures 1 and 2).

- *Left SLF1 and total empathy*: A negative association along the left SLF1 (11 points, 37.1 mm 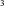; CWP =0.032; peak Z=−3.07), located in the posterior portion of the tract.
- *Left SLF3 and affective empathy*: The largest cluster, along the left SLF3 (15 points, 50.6 mm 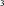; CWP =0.020; peak Z=−2.12).
- *Genu of the corpus callosum and total empathy*: A negative association in the genu (8 points, 27.0 mm 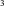; CWP =0.048; peak Z=−2.53).

**TABLE 2:** Along‐tract clusters showing negative FA–empathy associations at within‐tract permutation correction (5000 permutations). Cluster‐wise p‐values (CWP) are corrected within tract; none survived correction across all 168 analyses (q, Benjamini–Hochberg). Partial r (controlling for age and sex) and 95% CI are descriptive effect sizes for the cluster‐mean FA.

Tract	CASES‐K	Size	Size	Peak	CWP	FDR q	Partial r	95% CI
	Subscale	(pts)	(mm 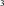 )	Z	(within)	(across 168)	(FA)	
lh.SLF3	Affective	15	50.6	−2.12	0.020	≈1.0	−0.60	[−0.81,−0.26]
lh.SLF1	Total	11	37.1	−3.07	0.032	≈1.0	−0.38	[−0.68,+0.02]
cc.Genu	Total	8	27.0	−2.53	0.048	≈1.0	−0.52	[−0.77,−0.15]

No clusters were found for the cognitive or somatic subscales in any tract.

#### 3.2.1 Correction Across the Full Analytic Space

When the most significant CWP from each of the 168 analyses was corrected across the entire search space, none of the three clusters remained significant under either Benjamini–Hochberg FDR (all q≈1.0) or Bonferroni correction (all $p_{\mathrm{adj}}=1.0$). The three associations are therefore reported as *exploratory and hypothesis‐generating* rather than as confirmed effects.

#### 3.2.2 Effect Sizes and Robustness

For descriptive purposes, the cluster‐mean FA partial correlations (controlling for age and sex) were r=−0.38 (95% CI [−0.68,+0.02]) for left SLF1 × total, r=−0.60 ([−0.81,−0.26]) for left SLF3 × affective, and r=−0.52 ([−0.77,−0.15]) for genu × total (Table 2). All three associations were essentially unchanged when average in‐scanner translation was added as a covariate (left SLF1 r=−0.46; left SLF3 r=−0.60; genu r=−0.52), indicating that they were not driven by head motion.

#### 3.2.3 Concordance With Other Diffusion Metrics

Within each FA‐defined cluster, the negative FA–empathy association was accompanied by *higher* RD with essentially *unchanged* AD; MD effects were intermediate (Table 3). These post‐hoc analyses share the FA cluster selection and are interpreted only as internal concordance checks (see Section 4).

**TABLE 3:** Concordance of diffusion metrics within the FA‐defined clusters: Partial correlations (controlling for age and sex) between each cluster‐mean metric and the relevant empathy score. The FA ↓/RD ↑/AD ≈ pattern is consistent across clusters. These are post‐hoc, non‐independent analyses (see text).

Tract	CASES‐K subscale	FA	MD	AD	RD
lh.SLF1	Total	−0.38	+0.36	+0.05	$+0.43^{*}$
lh.SLF3	Affective	$-0.60^{**}$	$+0.41^{*}$	+0.01	$+0.51^{*}$
cc.Genu	Total	$-0.52^{**}$	+0.28	−0.23	$+0.50^{*}$

Abbreviations: AD, axial diffusivity; FA, fractional anisotropy; MD, mean diffusivity; RD, radial diffusivity.

∗p<0.05.

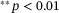
 (uncorrected, descriptive).

## 4 Discussion

In this exploratory study of 26 typically developing children, along‐tract analysis with within‐tract permutation correction identified three negative associations between FA and trait empathy: lower FA in the left SLF1 and the genu of the corpus callosum with higher total empathy, and lower FA in the left SLF3 with higher affective empathy. No associations were found for cognitive or somatic empathy. Critically, none of these clusters survived correction across the full set of 168 tract–subscale analyses; they are therefore best regarded as candidate relationships to be tested in larger samples, and the interpretations below are offered as hypotheses rather than conclusions.

### 4.1 SLF and Empathy

The two SLF associations were left‐lateralized and involved distinct subdivisions. The SLF is a major association tract connecting frontal and parietal cortices, subdivided into three branches with distinct trajectories (Makris et al. 2005): SLF1 connects superior parietal lobule with dorsal prefrontal cortex, SLF2 links angular gyrus with dorsolateral prefrontal cortex, and SLF3 extends from the supramarginal gyrus to ventral premotor and prefrontal regions. The SLF has been repeatedly implicated in empathy and social cognition (Wang et al. 2018; Veerareddy et al. 2025), although, as noted above, the direction of FA–empathy associations is inconsistent across studies, with both positive (Parkinson and Wheatley 2014; Veerareddy et al. 2025) and negative (Steinberg et al. 2022) relationships reported.

The localization of the affective‐empathy association to the left SLF3 is of interest because SLF3 connects the inferior frontal gyrus—a node of the putative mirror neuron system—with the inferior parietal lobule (Rizzolatti and Craighero 2004; Makris et al. 2005). The mirror neuron system has been proposed to support understanding of others’ actions and emotions through embodied simulation (Rizzolatti and Craighero 2004; Iacoboni and Dapretto 2006), and SLF3 could plausibly contribute to the simulation‐based component of affective empathy (Blair 2005). We emphasize, however, that this interpretation is speculative: the cluster did not survive whole‐analysis correction, the somatic subscale (also linked to motor mimicry) showed no association, and our data cannot adjudicate any specific mechanism. The apparent specificity to the affective subscale is consistent with—but by no means establishes—the theoretical distinction between cognitive perspective‐taking and affective resonance (Decety and Jackson 2004; Blair 2005).

### 4.2 Corpus Callosum Genu and Empathy

The genu, the most anterior callosal segment, carries fibers connecting bilateral prefrontal cortices (Paul et al. 2007), supporting interhemispheric integration relevant to social cognition (Wang and Olson 2018). Steinberg et al. (2022) likewise reported a negative FA–empathy association in the corpus callosum, and Comes‐Fayos et al. (2018) identified the callosum among tracts consistently implicated in empathy. Our genu finding is consistent with this literature but, given the small sample and lack of whole‐analysis significance, should be treated as preliminary.

### 4.3 Interpretation of the Negative Direction

All exploratory associations were negative, with higher empathy associated with lower FA—consistent with Steinberg et al. (2022) in young females but contrary to two adult studies reporting positive associations (Parkinson and Wheatley 2014; Veerareddy et al. 2025). Because FA is biologically nonspecific (see Section 1), a negative FA–behavior association carries no straightforward biological meaning; in particular, it does not imply “worse” white matter in more empathic children. With ∼90% of white matter voxels containing crossing fibers (Jeurissen et al. 2013), greater fiber complexity alone can lower FA even when individual fiber populations are intact.

Within our FA‐defined clusters, the negative FA effects were accompanied by higher RD with unchanged AD (Table 3)—a pattern often linked to lower myelination (Song et al. 2002), but equally attributable here to greater fiber dispersion or partial‐volume contributions in these crossing‐fiber‐rich pathways (Jeurissen et al. 2013; Jones et al. 2013). We therefore refrain from a myelination‐specific interpretation. Developmentally, white matter undergoes protracted maturation through childhood and adolescence (Lebel and Beaulieu 2012; Simmonds et al. 2014), and structure–behavior relationships in children need not match adult patterns; The negative direction could reflect features of an actively maturing system rather than reduced “integrity,” but this is speculative and only one of several candidate explanations. Finally, because affective empathy differed by sex in our sample and white matter maturation is itself sex‐dependent (Simmonds et al. 2014), sex may interact with these associations; our models controlled for sex, but the sample is too small to characterize sex interactions, which remain a question for larger studies.

### 4.4 Subscale Specificity

No associations emerged for cognitive or somatic empathy. Cognitive empathy may depend more on gray matter substrates (e.g., temporoparietal junction, medial prefrontal cortex) or on distributed connectivity less detectable at the single‐tract level, and somatic empathy may rely on shorter‐range connections not well captured by the long‐range pathways TRACULA reconstructs. These null results do not exclude white matter involvement in cognitive or somatic empathy; given the limited sample size, they could equally reflect insufficient statistical power, and should not be over‐interpreted as evidence of specificity.

### 4.5 Limitations

Several limitations temper these findings. First, the sample was small (N=26) and the analysis comprised 168 tract–subscale tests; although within‐tract permutation testing controlled error along each tract, no cluster survived correction across the full search space, so the results are exploratory and at elevated risk of being false positives. Second, the design was cross‐sectional and correlational, precluding any causal, maturational, or mechanistic inference; longitudinal data are needed to relate white matter change to empathy change. Third, although FA was complemented here by MD/AD/RD concordance analyses, all metrics are nonspecific, and advanced multi‐compartment or fixel‐based models (Genc et al. 2017) would better characterize the underlying microstructure. Fourth, the cluster‐mean correlations and scatterplots are non‐independent of cluster selection and are reported descriptively only. Fifth, the CASES‐K is a child self‐report measure subject to social‐desirability and insight limitations; multi‐method assessment would strengthen future work. Sixth, the 7–15‐year age span covers substantial maturation; although empathy scores were not significantly age‐related and all models controlled for age, larger samples are required to model age‐specific and potentially nonlinear effects.

## 5 Conclusion

In a small sample of typically developing children, this exploratory study identified candidate negative associations between trait empathy and FA in the SLF and the genu of the corpus callosum, with a tentative dissociation in which the left SLF3 related specifically to affective empathy. These associations did not survive correction across the full analytic space and are offered as hypotheses: They are consistent with the possibility that different empathy dimensions relate to partially distinct white matter pathways during development, but they require replication in larger, ideally longitudinal, samples before any maturational or mechanistic interpretation can be supported. More broadly, the results illustrate both the promise of combining automated tractography with multidimensional empathy measures in developmental neuroscience and the importance of cautious, appropriately corrected, and biologically circumspect interpretation of FA–behavior associations.

## Author Contributions

**June Christoph Kang**: conceptualization, investigation, funding acquisition, writing – original draft, writing – review and editing, visualization, validation, methodology, formal analysis, project administration, data curation, resources.

## Funding

This work was supported by the National Research Foundation of Korea under Grants NRF‐2018R1D1A1B07048820, NRF‐RS202300272647, and NRF‐RS202523524143.

## Ethics Statement

All procedures were approved by the Institutional Review Board of Korea University Guro Hospital (approval number: 2021GR0275) and were conducted in accordance with the Declaration of Helsinki.

## Consent

Written informed consent was obtained from a parent or legal guardian of every participant, and verbal assent was obtained from each child.

## Conflicts of Interest

The author declares no conflicts of interest.

## Data Availability

Processed, tract‐level summary data supporting these findings are available from the corresponding author upon reasonable request. Raw T1‐weighted and diffusion‐weighted images cannot be shared publicly because of privacy and ethical restrictions on pediatric neuroimaging data.
